# Family history of early onset acute lymphoblastic leukemia is suggesting genetic associations

**DOI:** 10.1038/s41598-021-90542-7

**Published:** 2021-06-11

**Authors:** Xinjun Li, Kristina Sundquist, Jan Sundquist, Asta Försti, Kari Hemminki

**Affiliations:** 1 Center for Primary Health Care Research, Lund University, Malmö, Sweden; 2 Department of Family Medicine and Community Health, Department of Population Health Science and Policy, Icahn School of Medicine at Mount Sinai, New York, NY USA; 3 Center for Community-Based Healthcare Research and Education (CoHRE), Department of Functional Pathology, School of Medicine, Shimane University, Matsue, Japan; 4 Hopp Children’s Cancer Center (KiTZ), Heidelberg, Germany; 5 Division of Pediatric Neurooncology, German Cancer Research Center (DKFZ), German Cancer Consortium (DKTK), Heidelberg, Germany; 6 Faculty of Medicine and Biomedical Center in Pilsen, Charles University in Prague, 30605 Pilsen, Czech Republic; 7 Division of Cancer Epidemiology, German Cancer Research Centre (DKFZ), 69120 Heidelberg, Germany

**Keywords:** Cancer, Oncology, Risk factors

## Abstract

Childhood acute lymphoblastic leukemia (ALL) has an origin in the fetal period which may distinguish it from ALL diagnosed later in life. We wanted to test whether familial risks differ in ALL diagnosed in the very early childhood from ALL diagnosed later. The Swedish nation-wide family-cancer data were used until year 2016 to calculate standardized incidence ratios (SIRs) for familial risks in ALL in three diagnostic age-groups: 0–4, 5–34 and 35 + years. Among 1335 ALL patients diagnosed before age 5, familial risks were increased for esophageal (4.78), breast (1.42), prostate (1.40) and connective tissue (2.97) cancers and leukemia (2.51, ALL 7.81). In age-group 5–34 years, rectal (1.73) and endometrial (2.40) cancer, myeloma (2.25) and leukemia (2.00, ALL 4.60) reached statistical significance. In the oldest age-group, the only association was with Hodgkin lymphoma (3.42). Diagnostic ages of family members of ALL patients were significantly lower compared to these cancers in the population for breast, prostate and rectal cancers. The patterns of increased familial cancers suggest that BRCA2 mutations could contribute to associations of ALL with breast and prostate cancers, and mismatch gene PMS2 mutations with rectal and endometrial cancers. Future DNA sequencing data will be a test for these familial predictions.

## Introduction

Acute lymphoblastic leukemia (ALL) is together with nervous system cancer the most common childhood malignancy^1^. ALL has been earlier considered largely a non-hereditary disease but with large sequencing efforts growing numbers of predisposing genetic alterations have been revealed^2,3^. The implicated variants are very rare but they are involved in some key functional pathways, such as DNA repair, immunodeficiency, tumor suppression and Down syndrome^2,4^. The recent review by Pui and coworkers listed 13 syndromes associated with the risk of ALL; of these, the Fanconi anemia group included 21 individual genes, such as BRCA2^2^. While many of these predisposing genes are shared by other hematological malignancies, some are also implicated in solid tumors, including ATM, BLM, Fanconi anemia genes, TP53 (Li-Fraumeni syndrome) and NF1^2^. The largest number of childhood leukemias with known germline mutations are manifested in the Li-Fraumeni syndrome and most of these are ALL^3,5^. Even though DNA repair genes are prominently present among the predisposing genes, the important group of mismatch repair genes is only represented through the very rare constitutional mismatch deficiency syndrome, manifesting many types of cancers including ALL^2,6^. In addition to these rare high-risk predisposing genes, genome-wide association studies (GWASs) have identified increasing numbers of common low-risk gene variants in the germline^7–9^. Jointly these are estimated to account for 21% of heritability in ALL^8^. Familial risk has been described for ALL and it is particular high among monozygotic twins where sharing of blood cells during pregnancy has been explained as the mechanism^10–12^. In spite of sharing of many predisposing genes between ALL and solid tumors, population-level family studies have not been able to demonstrate associations of ALL with other hematological or solid tumors, with the exception of testicular cancer^10,13^. In adult hematological malignancies familial sharing has been shown between different types^14,15^.

The peak incidence of ALL occurs within the first years after birth which has been suggested to indicate the origin of this disease is in the fetal period^1,16,17^. The two-hit theory was developed through observations from twin births as reviewed by Greaves and coworkers^18,19^. The first hit takes place in utero in one of the twin pairs who transmits the mutant clone to the co-twin. The second hit takes place postnatally and independently in the twin pairs and may be genetic or involve contribution of immune disturbances and other environmental factors^20^. These data show that the developmental timing is etiologically important for ALL and it has implication for treatment and prognosis^1,16,17^. We therefore wanted to consider family history of ALL with any other cancers according to age at ALL diagnosis, in early and late childhood compared to old age using the most recent update of the Swedish Family-Cancer Database.

## Results

The characteristics of the study population are shown in Table 1. The total index population in the offspring generations amounted to 9.3 million individuals recorded from year 1932 onwards; the total population, including the parental generation was16.8 million. ALL was diagnosed in 3724 individuals, 56.3% males and 43.7% females. Diagnostic age-group 0–4 years included 35.8%, 5–34 years 50.3% and 35 + years 13.8% of ALL. The age- and sex-specific incidence of ALL is also shown in in Table 1. The rate of 4.4/100,000 is noticed among the very young, somewhat higher among males, as in all age groups.

**Table 1:** Characteristics of study population, number of cases and incidence rate (IR, per 100 000 person years) of acute lymphoblastic leukemia (ALL) in Sweden, 1958–2016.

	Men			Women			All		
	No	%	IR (per 100 000 person years)	No	%	IR (per 100 000 person years)	No	%	IR (per 100 000 person years)
Population,	4,792,416	51.3		4,546,466	48.7		9,338,882		
Cases, ALL	2098	56.3	1.3	1626	43.7	1.1	3724		1.2
**Age at diagnosis (years)**									
0–4	697	33.2	4.5	638	39.2	4.4	1335	35.8	4.4
5–34	1117	53.2	1.3	758	46.6	0.9	1875	50.4	1.1
35 +	284	13.6	0.5	230	14.2	0.4	514	13.8	0.5

Familial risks for ALL were assessed when family members were diagnosed with any cancer according to ALL diagnostic age (Table 2). The overall risk was significant in the youngest (1.37) and the middle (1.18) age-groups. The individual cancers with significant associations with ALL diagnosed at age 0–4 years were esophageal (4.78), breast (1.42), prostate (1.40) and connective tissue (2.97) cancers and leukemia (2.51, particularly ALL 7.81). In age-group 5–34 years, rectal (1.73), endometrial (2.40) and prostate cancers (1.27), myeloma (2.25) and leukemia (2.00, ALL 4.60) reached statistical significance. In the oldest age-group, the only association was with Hodgkin lymphoma (3.42).

**Table 2:** Familial risks for acute lymphoblastic leukemia diagnosed at different ages.

Cancer in any family members	Age at diagnosis (years)															
	0–4				5–34				35 +				All			
	O	SIR	95% CI		O	SIR	95% CI		O	SIR	95% CI		O	SIR	95% CI	
Upper aerodigestive tract	8	2.06	0.88	4.07	9	1.02	0.46	1.94	8	1.77	0.76	3.51	25	1.45	0.94	2.14
Esophagus	5	**4.78**	**1.51**	**11.25**	3	1.08	0.20	3.20	1	0.59	0.00	3.35	9	1.63	0.74	3.10
Stomach	1	0.39	0.00	2.25	10	1.35	0.64	2.49	10	1.40	0.67	2.58	21	1.23	0.76	1.88
Colon	11	1.22	0.61	2.19	23	1.01	0.64	1.52	11	0.69	0.34	1.24	45	0.94	0.69	1.26
Rectum	10	1.67	0.79	3.07	26	**1.73**	**1.13**	**2.54**	12	1.22	0.63	2.13	48	**1.55**	**1.15**	**2.06**
Liver	2	0.74	0.07	2.72	9	1.23	0.56	2.34	4	0.69	0.18	1.80	15	0.95	0.53	1.57
Pancreas	3	1.03	0.19	3.05	11	1.37	0.68	2.47	5	0.82	0.26	1.92	19	1.12	0.67	1.75
Lung	15	1.56	0.87	2.57	35	1.26	0.88	1.76	15	0.81	0.45	1.34	65	1.16	0.90	1.48
Breast	45	**1.42**	**1.04**	**1.91**	62	0.89	0.68	1.15	35	1.02	0.71	1.42	142	1.05	0.88	1.24
Cervix	4	0.85	0.22	2.20	9	0.93	0.42	1.77	4	0.84	0.22	2.16	17	0.89	0.52	1.42
Endometrium	8	2.30	0.98	4.55	25	**2.40**	**1.55**	**3.55**	9	1.06	0.48	2.03	42	**1.88**	**1.35**	**2.54**
Ovary	2	0.62	0.06	2.28	10	1.20	0.57	2.22	11	1.81	0.90	3.26	23	1.31	0.83	1.96
Prostate	44	**1.40**	**1.01**	**1.88**	104	**1.27**	**1.04**	**1.54**	46	0.88	0.64	1.17	194	**1.17**	**1.01**	**1.35**
Testis	7	1.66	0.66	3.45	9	1.42	0.65	2.72	0				16	1.36	0.78	2.21
Kidney	4	0.84	0.22	2.17	10	0.81	0.38	1.49	9	0.95	0.43	1.82	23	0.86	0.55	1.30
Urinary bladder	10	1.48	0.71	2.74	11	0.56	0.28	1.00	18	1.10	0.65	1.74	39	0.91	0.65	1.25
Melanoma	23	1.50	0.95	2.26	36	1.19	0.83	1.65	12	0.92	0.47	1.61	71	1.21	0.95	1.53
Skin	6	1.08	0.39	2.36	22	1.31	0.82	1.99	14	0.84	0.46	1.41	42	1.08	0.77	1.45
Nervous system	9	0.79	0.36	1.50	24	1.03	0.66	1.53	18	1.61	0.95	2.55	51	1.11	0.83	1.46
Thyroid gland	6	1.63	0.59	3.56	4	0.58	0.15	1.49	3	1.00	0.19	2.95	13	0.95	0.51	1.64
Endocrine glands	4	0.76	0.20	1.97	9	0.77	0.35	1.46	11	1.66	0.83	2.99	24	1.02	0.65	1.51
Connective tissue	6	**2.97**	**1.07**	**6.50**	5	1.18	0.37	2.77	2	0.83	0.08	3.04	13	1.50	0.79	2.57
Hodgkins disease	4	1.66	0.43	4.29	7	1.65	0.65	3.42	6	**3.42**	**1.23**	**7.50**	17	**2.02**	**1.18**	**3.24**
Non-Hodgkins lymphoma	11	1.48	0.73	2.65	21	1.15	0.71	1.77	16	1.32	0.75	2.14	48	1.27	0.94	1.68
Myeloma	1	0.46	0.00	2.62	14	**2.25**	**1.22**	**3.78**	7	1.25	0.50	2.59	22	1.57	0.98	2.38
Leukemia	20	**2.51**	**1.53**	**3.89**	36	**2.00**	**1.40**	**2.78**	11	0.94	0.47	1.69	67	**1.78**	**1.38**	**2.26**
Acute lymphatic leukemia	13	**7.81**	**4.14**	**13.40**	11	**4.60**	**2.28**	**8.26**	0				24	**5.25**	**3.36**	**7.83**
Chronic lymphatic leukemia	0				7	1.49	0.59	3.09	3	0.74	0.14	2.19	10	0.96	0.46	1.78
Acute myeloid leukemia	0				6	1.88	0.68	4.13	3	1.33	0.25	3.93	9	1.32	0.60	2.53
Chronic myeloid leukemia	1	1.25	0.00	7.19	2	1.19	0.11	4.37	2	1.96	0.18	7.21	5	1.43	0.45	3.36
Primary unknown	2	0.64	0.06	2.34	8	0.95	0.41	1.88	12	**1.98**	**1.02**	**3.47**	22	1.25	0.78	1.89
All	273	**1.37**	**1.21**	**1.55**	560	**1.18**	**1.08**	**1.28**	315	1.06	0.94	1.18	1148	**1.18**	**1.11**	**1.25**

Bolding indicates that 95% CIs do not include 1.00 (result is significant).

O, Observed; SIR, Standardized incidence ratio; CI, Confidence intervals.

Familial associations of female ALL patients are shown in Supplementary Table 1. The association with endometrial cancer was significant in the two youngest age groups, which also showed novel associations with testicular cancer (3.01 and 2.85). The association with prostate cancer was shifted from the youngest only to the middle age-group (compared to Table 2). The oldest age-group showed a novel association with upper aerodigestive tract cancer (3.16) and the association with rectal cancer was now in this age-group compared to the middle age-group in Table 2. The female ALL population included 3 pairs of twins which explains the high ALL risk. Analysis of ALL risks in male patients showed no new associations (data not shown).

We searched for more details about diagnostic ages of the family members of ALL patients with significant associations, collected from Table 2. There were 45 female probands diagnosed with breast cancer; their mean diagnostic age was 50.5 years (Table 3). In the Swedish female population 53,677 breast cancers were diagnosed between 2010 and 2016; their mean diagnostic age was 64.1 years (*p* ≤ 0.001 to ALL probands). In similar comparisons for prostate, connective tissue, rectal and endometrial cancers the diagnostic age of the ALL probands was lower than the correspondence ages in the population but the difference was significant only for prostate and rectal cancers.

**Table 3:** Mean diagnostic age in cancers in probands of ALL families and in the population.

	Probands in ALL families (N)	Population with the same cancer (N)	
Cancer	Mean diagnostic age		*P*-value
Breast	50.5 ± 11.0 (45)	64.1 ± 13.7 (53,677)	< 0.001
Prostate	63.1 ± 9.1 (44)	69.3 ± 8.9 (70,404)	0.005
Connective tissue	49.0 ± 16.9 (6)	62.2 ± 20.3 (2,192)	0.33
Rectum	59.7 ± 12.4 (48)	69.8 ± 11.9 (14,262)	< 0.001
Endometrium	62.6 ± 9.8 (42)	69.3 ± 11.4 (10,480)	0.38

## Discussion

Familial association of ALL with ALL is known even among singleton siblings, and the association of testicular cancer has been reported before^10,12^. The novel associations were to some extent depending on the age at ALL diagnosis; the youngest patients (accounting 35. 8% of ALL) had novel familial associations with esophageal, breast and prostate cancers; the diagnostic group of 5–34 years showed associations with rectal and endometrial cancers and with myeloma; the oldest age group had an association with Hodgkin lymphoma. However, none of the age-group related association were significantly different between the age-groups (i.e., their 95%CIs overlapped) so we cannot claim that the associations were age-group specific with the current case numbers. Female ALL patients showed a novel association with upper aerodigestive tract cancer, in addition to the known risk with testicular cancer. The spectrum of familial cancers associated with ALL appears heterogeneous but we try and see if anything beyond chance associations can be found. This is done in terms of consistency of the findings in this study and consistency with genetic studies from the literature. With such a convention, we do not discuss further solitary association, such female associations with upper aerodigestive tract cancer.

Breast cancer was one of the associated cancers in families of very young ALL patients, and the Fanconi anemia-BRCA2 pathway is a known risk factor for childhood ALL^2,21^. However, if BRCA2 was involved one would expect that ovarian cancer risk was also increased. The reason why this was not the case could be that BRCA2 mutations are associated with far lower risk of ovarian than breast cancer^22^. BRCA2 mutations also predispose to prostate cancer which was also associated with ALL^22^. The younger diagnostic age of breast and prostate cancer in ALL families supported the suggested involvement of BRCA2.

ALL patients showed a familial association with rectal and endometrial cancers. With colorectal cancer, endometrial cancer is a signal malignancy for mismatch repair (MMR) defects in Lynch syndrome^23^. The dilemma is that hematopoietic malignancies are not considered part of this syndrome and, for ALL, the MMR genes and particularly the PMS2 gene are only playing a role in the very rare constitutional mismatch deficiency syndrome, which occasionally manifests ALL^2,6^. The syndrome is most common in inbred populations, unlike the Swedish one^24^. PMS2 mutations have been rare in Lynch syndrome, probably due to difficulties in their identification, but more recently these have been reported, predisposing to endometrial cancer with a relative risk of about twice higher than for colorectal cancer^23,25–27^. Recently, PMS2 mutations have been reported also in childhood ALL^21,28^. Thus we hypothesize that the associations of ALL with endometrial and rectal cancer may be explained by germline variants in the PMS2 gene. These mutations are not associated with ovarian cancer, another cancer manifesting in Lynch syndrome, and not increased in the present analysis^29^. Early onset of ALL associated rectal cancer was in line with Lynch syndrome.

The association with connective tissue cancer could suggest an underlining Li-Fraumeni syndrome but with 6 cases no further evidence can be obtained even though the age of onset in relatives was 13 years below the population age of onset for this cancer^2,30^. Association with myeloma and Hodgkin lymphoma could be plausible in terms of all being hematological neoplasms of B-cell origin although previous data for myeloma are lacking^15^. ALL and Hodgkin lymphoma share susceptibility to ATM and NBN mutations^2^. The previously described association with testicular cancer was suggested to be related to the origin of testicular cancer in fetal gonocytes and shared gestational mechanistic with ALL^31,32^. The individual associations of ALL with esophageal and upper aerodigestive tract cancers and with cancer of unknown primary lack known links to ALL and may be fortuitous findings.

Even in the expanded version the present ALL family database is small due to the rareness of ALL whereby statistical power is limited. As the limitation is universal and the possibility to conduct family studies is also internationally limited, it is hardly likely that literature support to the present results could be found. However, the increasing in large-scale sequencing efforts of DNA from childhood cancer patients will offer a possibility to verify or refute the current proposals about the possible contributing germline variants.

In conclusion, all previous associations of childhood ALL with other familial cancers have originated from the earlier version of the present research dataset, while a US study was negative^10,33^. In the present analysis we expanded the findings and suggest that BRCA2 could explain the association of early onset ALL with breast and prostate cancers. For the breast cancer association other rare predisposing genes, such as Fanconi anemia, ATM and BLM could contribute^2^. While the association with endometrial and rectal cancers suggest involvement of Lynch syndrome, the specific mismatch repair gene could be PSM2. Although the present results support the notion that ALL may not be only a sporadic disease, there is nevertheless a need to verify these results from independent sources.

## Materials and methods

Family relationships were obtained from the Multigeneration Register, containing the Swedish population in families. ‘The offspring generations’ were born after 1931 and by year 2016 oldest offspring reached age 84 years; siblings could be defined only in the offspring generations. Cancers were identified from the Swedish Cancer Registry which was started in 1958 using codes of the International Classification of Diseases version 7 and later revisions. Information from the registers was linked at the individual level via the national 10-digit civic registration number. In the linked dataset, civic registration numbers were replaced with serial numbers to ensure anonymity.

Familial risk was considered for offspring with childhood leukemia whose first-degree relatives (parent or siblings) were diagnosed with any cancer; these were thus probands and no more distant relatives were included. Standardized incidence ratios (SIRs) were calculated as the ratio of observed to expected number of cases. The expected numbers were calculated for all individuals without cancer in family members, and the rates were standardized by 5-year-age, gender, period (5 year age-group), socioeconomic status and residential area. The expected rates were derived from the present dataset covering the Swedish population. The 95% confidence interval (95%CI) of the SIR was calculated assuming a Poisson distribution. Observed cases (O) indicate the persons whom the SIR was calculated. In presenting results only statistically significant results (95%CI did not include 1.00) were called even though the significance is not repeated in text.

In the comparison of diagnostic ages of probands in the families of ALL patients to the diagnostic ages in the population we limited the population to persons diagnosed between years 2010 and 2016, which we estimated to be the diagnostic period of most family members of ALL patients. Among ALL probands we selected the age group for which the statistical significance was found in order not to weaken the likelihood of finding a difference. The exceptions were rectal and endometrial cancers for which the SIR for ‘All’ diagnostic age groups was clearly significant.

In assessing the results we considered the large number of associations that were generated with all cancers and the unavoidable likelihood of false positive results. Some formal approaches, such as the Bonferroni correction, are not suitable for this kind of data for which a priori power of detection is low^34^. In discussion of the findings, we search support from two or more positive familial associations or from literature describing similar associations or relevant germline mutation data.

### Ethical statement

The study was conducted in accordance with the tenets of the Declaration of Helsinki. We have ethical approval (February 6, 2013) for this study from the Regional Ethical Review Board of Lund University (Dnr 2012/795). Patient consent was not needed as the study used only de-identified registry based secondary data.

## Supplementary Information

Supplementary Table.

## Data Availability

The data that support the findings of this study are available from Lund University but restrictions apply to the availability of these data, which were used under license for the current study and so are not publicly available.
