# Supplementary material for: Family history of early onset acute lymphoblastic leukemia is suggesting genetic associations
Source: Sci Rep. 2021 Jun 11;11:12370. doi: 10.1038/s41598-021-90542-7 (PMC8195979; doi:10.1038/s41598-021-90542-7)
Supplement: Supplementary file 1 — Supplementary Table. [file 41598_2021_90542_MOESM1_ESM.docx]

FAMILY HISTORY OF EARLY ONSET ACUTE LYMPHOBLASTIC LEUKEMIA IS SUGGESTING GENETIC ASSOCIATIONS

Xinjun Li ^1^, Kristina Sundquist ^1,2,3^, Jan Sundquist^1,2,3^, Asta Försti^1,4,5^*, Kari Hemminki^1,6,7^*

Familial associations of female ALL patients are shown in **Supplementary Table 1**. The association with endometrial cancer was significant in the two youngest age groups, which also showed novel associations with testicular cancer (3.01 and 2.85). The oldest age-group showed a novel association with upper aerodigestive tract cancer (3.16). The association with prostate and rectal cancers were also observed.

| **Supplementary Table 1. Familial risks for female acute lymphoblastic leukemia patients diagnosed at different ages.** | | | | | | | | | | | | | | | | | | |  |
| --- | --- | --- | --- | --- | --- | --- | --- | --- | --- | --- | --- | --- | --- | --- | --- | --- | --- | --- | --- |
|  | Age at diagnosis (years) | | | | | | | | | | | | | |  |  |  |  |  |
|  | 0-4 | | | |  | 5-34 | | | |  | 35 + | | | |  | All | | | |
| Cancer in any family members | O | SIR | 95% CI | |  | O | SIR | 95% CI | |  | O | SIR | 95% CI | |  | O | SIR | 95% CI | |
| Upper aerodigestive tract | 4 | 2.21 | 0.58 | 5.72 |  | 3 | 0.87 | 0.16 | 2.58 |  | 6 | **3.16** | **1.14** | **6.92** |  | 13 | 1.82 | 0.96 | 3.12 |
| Stomach | 1 | 0.86 | 0.00 | 4.93 |  | 1 | 0.35 | 0.00 | 1.99 |  | 6 | 1.94 | 0.70 | 4.25 |  | 8 | 1.12 | 0.48 | 2.22 |
| Colon | 1 | 0.24 | 0.00 | 1.38 |  | 8 | 0.90 | 0.39 | 1.79 |  | 3 | 0.43 | 0.08 | 1.28 |  | 12 | 0.60 | 0.31 | 1.05 |
| Rectum | 5 | 1.83 | 0.58 | 4.30 |  | 8 | 1.38 | 0.59 | 2.72 |  | 10 | **2.33** | **1.11** | **4.31** |  | 23 | **1.79** | **1.13** | **2.69** |
| Liver | 1 | 0.82 | 0.00 | 4.68 |  | 4 | 1.41 | 0.37 | 3.63 |  | 2 | 0.80 | 0.08 | 2.94 |  | 7 | 1.07 | 0.42 | 2.21 |
| Pancreas | 2 | 1.52 | 0.14 | 5.57 |  | 3 | 0.97 | 0.18 | 2.86 |  | 3 | 1.12 | 0.21 | 3.33 |  | 8 | 1.13 | 0.48 | 2.23 |
| Lung | 6 | 1.39 | 0.50 | 3.06 |  | 17 | 1.59 | 0.92 | 2.55 |  | 5 | 0.64 | 0.20 | 1.51 |  | 28 | 1.23 | 0.82 | 1.78 |
| Breast | 22 | 1.49 | 0.93 | 2.26 |  | 25 | 0.92 | 0.59 | 1.35 |  | 16 | 1.07 | 0.61 | 1.75 |  | 63 | 1.11 | 0.85 | 1.42 |
| Cervix | 2 | 0.91 | 0.09 | 3.34 |  | 3 | 0.79 | 0.15 | 2.34 |  | 1 | 0.50 | 0.00 | 2.85 |  | 6 | 0.75 | 0.27 | 1.64 |
| Endometrium | 5 | **3.22** | **1.02** | **7.57** |  | 9 | **2.23** | **1.01** | **4.25** |  | 5 | 1.36 | 0.43 | 3.20 |  | 19 | **2.05** | **1.23** | **3.21** |
| Ovary | 1 | 0.67 | 0.00 | 3.85 |  | 3 | 0.92 | 0.17 | 2.73 |  | 6 | 2.29 | 0.82 | 5.02 |  | 10 | 1.36 | 0.65 | 2.51 |
| Prostate | 19 | 1.32 | 0.79 | 2.07 |  | 49 | **1.54** | **1.14** | **2.03** |  | 18 | 0.79 | 0.47 | 1.25 |  | 86 | **1.25** | **1.00** | **1.54** |
| Testis | 6 | **3.01** | **1.08** | **6.60** |  | 7 | **2.85** | **1.13** | **5.90** |  | 0 |  |  |  |  | 13 | **2.62** | **1.39** | **4.48** |
| Kidney | 2 | 0.93 | 0.09 | 3.43 |  | 3 | 0.63 | 0.12 | 1.86 |  | 3 | 0.73 | 0.14 | 2.17 |  | 8 | 0.73 | 0.31 | 1.44 |
| Urinary bladder | 3 | 0.99 | 0.19 | 2.93 |  | 5 | 0.65 | 0.21 | 1.54 |  | 8 | 1.13 | 0.48 | 2.24 |  | 16 | 0.90 | 0.51 | 1.47 |
| Melanoma | 12 | 1.67 | 0.86 | 2.93 |  | 17 | 1.44 | 0.84 | 2.31 |  | 5 | 0.88 | 0.28 | 2.06 |  | 34 | 1.38 | 0.95 | 1.93 |
| Skin | 6 | 2.39 | 0.86 | 5.23 |  | 9 | 1.39 | 0.63 | 2.66 |  | 8 | 1.09 | 0.47 | 2.16 |  | 23 | 1.41 | 0.89 | 2.12 |
| Nervous system | 5 | 0.94 | 0.30 | 2.22 |  | 15 | 1.65 | 0.92 | 2.73 |  | 8 | 1.66 | 0.71 | 3.28 |  | 28 | 1.46 | 0.97 | 2.11 |
| Thyroid gland | 3 | 1.72 | 0.33 | 5.10 |  | 0 |  |  |  |  | 2 | 1.52 | 0.14 | 5.59 |  | 5 | 0.87 | 0.27 | 2.04 |
| Endocrine glands | 1 | 0.41 | 0.00 | 2.34 |  | 5 | 1.08 | 0.34 | 2.53 |  | 4 | 1.40 | 0.36 | 3.62 |  | 10 | 1.00 | 0.48 | 1.85 |
| Connective tissue | 2 | 2.14 | 0.20 | 7.88 |  | 3 | 1.85 | 0.35 | 5.47 |  | 0 |  |  |  |  | 5 | 1.39 | 0.44 | 3.26 |
| Hodgkins disease | 2 | 1.77 | 0.17 | 6.51 |  | 2 | 1.24 | 0.12 | 4.56 |  | 3 | 3.99 | 0.75 | 11.80 |  | 7 | 2.00 | 0.79 | 4.15 |
| Non-Hodgkins lymphoma | 4 | 1.18 | 0.31 | 3.06 |  | 12 | 1.71 | 0.88 | 3.00 |  | 8 | 1.51 | 0.65 | 3.00 |  | 24 | 1.53 | 0.98 | 2.28 |
| Myeloma | 0 |  |  |  |  | 5 | 2.07 | 0.65 | 4.87 |  | 5 | 2.02 | 0.64 | 4.74 |  | 10 | 1.70 | 0.81 | 3.14 |
| Leukemia | 12 | **3.32** | **1.70** | **5.81** |  | 17 | **2.45** | **1.43** | **3.94** |  | 5 | 0.98 | 0.31 | 2.31 |  | 34 | **2.17** | **1.50** | **3.04** |
| Acute lymphatic leukemia | 10 | **12.74** | **6.07** | **23.52** |  | 5 | **5.48** | **1.73** | **12.89** |  | 0 |  |  |  |  | 15 | **7.85** | **4.38** | **12.98** |
| Chronic lymphatic leukemia | 0 |  |  |  |  | 3 | 1.64 | 0.31 | 4.87 |  | 1 | 0.56 | 0.00 | 3.21 |  | 4 | 0.92 | 0.24 | 2.38 |
| Acute myeloid leukemia | 0 |  |  |  |  | 5 | **4.09** | **1.29** | **9.62** |  | 0 |  |  |  |  | 5 | 1.77 | 0.56 | 4.16 |
| Chronic myeloid leukemia | 0 |  |  |  |  | 1 | 1.51 | 0.00 | 8.66 |  | 2 | 4.57 | 0.43 | 16.80 |  | 3 | 2.05 | 0.39 | 6.08 |
| Primary unknown | 1 | 0.73 | 0.00 | 4.16 |  | 2 | 0.63 | 0.06 | 2.30 |  | 5 | 1.89 | 0.59 | 4.43 |  | 8 | 1.11 | 0.47 | 2.19 |
| All | 131 | **1.43** | **1.20** | **1.70** |  | 240 | **1.30** | **1.14** | **1.47** |  | 147 | 1.14 | 0.96 | 1.34 |  | 518 | **1.28** | **1.17** | **1.39** |
| O=Observed; SIR=Standardized incidence ratio; CI=Confidence intervals. Bolding shows that 95%CI does not cover SIR 1.00. | | | | | |  |  |  |  |  |  |  |  |  |  |  |  |  |  |
